# Proposing an Ecosystem of Digital Health Solutions for Teens With Chronic Conditions Transitioning to Self-Management and Independence: Exploratory Qualitative Study

**DOI:** 10.2196/10285

**Published:** 2018-09-06

**Authors:** Emre Sezgin, Monica Weiler, Anthony Weiler, Simon Lin

**Affiliations:** ^1^ Research Information Solutions and Innovation The Research Institute Nationwide Children's Hospital Columbus, OH United States; ^2^ Stratos Innovation Group Columbus, OH United States

**Keywords:** chronic disease, chronic disease management, digital health, ecosystem, qualitative research, self-management, transition to independence, technology-based solutions

## Abstract

**Background:**

Chronic disease management is critical to quality of life for both teen patients with chronic conditions and their caregivers. However, current literature is largely limited to a specific digital health tool, method, or approach to manage a specific disease. Guiding principles on how to use digital tools to support the transition to independence are rare. Considering the physiological, psychological, and environmental changes that teens experience, the issues surrounding the transition to independence are worth investigating to develop a deeper understanding to inform future strategies for digital interventions.

**Objective:**

The purpose of this study was to inform the design of digital health solutions by systematically identifying common challenges among teens and caregivers living with chronic diseases.

**Methods:**

Chronically ill teens (n=13) and their caregivers (n=13) were interviewed individually and together as a team. Verbal and projective techniques were used to examine teens’ and caregivers’ concerns in-depth. The recorded and transcribed responses were thematically analyzed to identify and organize the identified patterns.

**Results:**

Teens and their caregivers identified 10 challenges and suggested technological solutions. Recognized needs for social support, access to medical education, symptom monitoring, access to health care providers, and medical supply management were the predominant issues. The envisioned ideal transition included a 5-component solution ecosystem in the transition to independence for teens.

**Conclusions:**

This novel study systematically summarizes the challenges, barriers, and technological solutions for teens with chronic conditions and their caregivers as teens transition to independence. A new solution ecosystem based on the 10 identified challenges would guide the design of future implementations to test and validate the effectiveness of the proposed 5-component ecosystem.

## Introduction

### Background

Self-management for those with chronic diseases is a significant component for maintaining wellbeing. Nationally, chronic diseases cause 7 out of 10 deaths of US citizens annually, and a large portion of national health care costs are generated by patients with chronic disease [1]. Hence, self-management sustainability greatly impacts national health care cost reductions while increasing the individual’s wellbeing and financial independence. Digital solutions developed in recent years assist patients in self-management. Youth especially use health care management technologies (eg, telehealth and mobile health [mHealth]), enabling greater self-management [2].

### Digital Solutions in Chronic Disease Management

Digital solutions and mHealth apps concerning chronic disease management (CDM) and behavioral change for teens have been widely discussed, with new solutions proposed in the literature. Hamine et al’s [3] review on the self-management of diabetes, cardiovascular disease, and chronic lung diseases concluded that mHealth could potentially facilitate adherence to CDM. Fedele et al [4] argued that mHealth interventions are also a viable approach in behavioral change interventions in young populations (<18 years). However, adopting technology to assist teens in transition is a work-in-progress. Huang et al [5], tested a Web-based and short message service (SMS) text message-delivered disease management app and reported that technology could be a useful and cost-effective solution as a transition intervention. They also discussed that the use of communication technologies (ie, mobile phone calls, SMS, email, and Voice over Internet Protocol) promoted engagement, relationship, and trust between teens and health care providers [6]. These studies support the use of digital solutions and demonstrate a promising next step in CDM technology. In another case, Holtz et al [7] developed and tested a patient-centered mobile app using focus group interviews with teens having type 1 diabetes (T1D) and their parents. Participants reported that they thought that the mobile app would help to improve communication among family members. Many of these studies focused on a specific delivery modality of digital interventions. Our study, in contrast, started with a broader inquiry into patient-centered needs and then explored relevant technological solutions.

Given the physiological, psychological, and environmental changes that teens experience in CDM, digital solutions may fail to keep up with expectations. Slater et al [2] underlined that mHealth interventions have failed to integrate into real-world settings and adoption practices. The impact of using self-management digital communication tools on relationships among parents, teens, and health care providers (HCPs) is unclear [8]. Furthermore, the evidence in the literature is limited regarding caregivers and teens in transition to independence, with no identified studies on digital communication between caregivers and teens in transition [8].

To improve the delivery of health care among teens transitioning to independence with chronic illnesses, Nationwide Children’s Hospital (NCH), OH, USA, undertook a quality improvement project. We employed a patient-centered approach to identify and better understand the core problems of CDM and to seek solutions to permit the teen to transition to independence. Our specific aim was to generate a roadmap for chronically ill teens to gain independence.

## Methods

### Study Design

Data collection methodology included a co-design framework with the use of generative tools utilizing verbal and projective techniques to collect rich data from participants regarding their needs and expectations [9,10]. Our study questions focused on “What common challenges do teens and their caregivers face in preparation for the transition to independent health management?” and “What digital solutions and opportunities would help to overcome the challenges and barriers?” To address these questions, we first identified the challenges, barriers, and gaps during the life journey, and then, we envisioned digital solutions and opportunities to facilitate a successful transition to independence and self-management. Throughout the interviews, participants shared their needs and concerns with the research team. Two of the coauthors (MW and AW) took the lead in interviews and analysis.

### Participants

An independent recruiting agency selected participants based on the following inclusion criteria: (1) age 13-18 years, (2) a minimum of one chronic condition for >6 months, and (3) medication taken multiple times a day. The NCH patient network also supported this recruitment process. During a telephone invitation, patients and caregivers were informed on voluntary participation, study goals, and financial compensations for their time. Subsequent interviews were held on NCH’s main campus. Parents signed a written consent form indicating their agreement to participate for themselves and their teen. The study was approved by NCH as a quality improvement project and was not subject to the Institutional Review Board.

The study enrolled 13 teens with chronic conditions and their caregivers (n=13). Patient ages ranged from 13 to 18 years. Chronic conditions included at least one of the following diseases: T1D, cystic fibrosis (CF), epilepsy, and attention deficit hyperactivity disorder (ADHD). Most participants had lived with the chronic condition for >5 years (Table 1).

### Data Collection

The aim was to develop an in-depth understanding of each participant’s experience starting from prediagnosis. Each interview commenced with a brief introduction of the study, including the goals, the agenda, and the roles.

**Figure 1:**
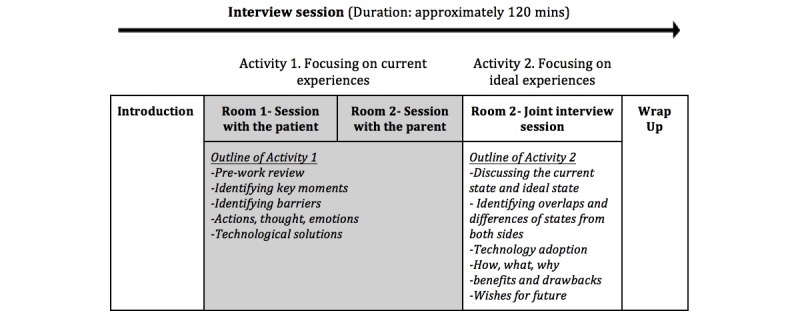
Outlining the interview process.

The first activity began with a review of the homework assignment. This was a short reflective exercise completed prior to the interviews including questions with visual illustrations about personality, people who they care about, personal environment, their typical day, and future self (Multimedia Appendix 1). To encourage free expression, concurrent sessions were scheduled for this first activity, separating teens and their parents (Figure 1).

**Table 1:** Patient demographic information (N=13).

Demographics		n (%)
**Age (years)**		
	11-13	2 (15)
	14-15	4 (31)
	16-18	7 (54)
**Gender**		
	Male	6 (46)
	Female	7 (54)
**Chronic condition**		
	Type 1 diabetes	4 (31)
	Cystic fibrosis	2 (15)
	Epilepsy	3 (23)
	Attention deficit hyperactivity disorder	4 (31)
**Length of time since diagnosis**		
	6 months-2 years	1 (8)
	2-5 years	1 (8)
	5-10 years	9 (69)
	>10 years	2 (15)

Next, the teen and caregiver worked together to envision an ideal journey that depicted the teen’s successful transition to independent management of their therapy. To promote their participation, a canvas (a large white page with visual guidelines) outlining the stages and tools was used, on which participants were able to follow the process as well as to type, draw, and elaborate their opinions (Multimedia Appendix 1).

The second activity was a joint session with the teen and caregiver. Together, each pair shared their current experience and ideal state and identified overlapping experiences and differences, technology adoptions, challenges, opportunities, and expectations in line with the transitioning. An additional canvas was presented to be used for outlining the ideal transition journey (Multimedia Appendix 1). The second session, which focused on technological solutions, discussed the use of technology apps in care, how they are used, their benefits and drawbacks, what the apps were that they wished for, what is needed, and why they were not using the technology. The interview session ended with a closure talk and soliciting final thoughts from the participants.

At least two researchers attended each session, which was recorded. Researchers served as a moderator or an assistant and a note taker. All recordings were transcribed and aggregated with memos and observational notes. Information on the canvases, recordings, and notes was analyzed by thematic analysis. The combination of information sources from both teens and caregivers increased the richness of the data acquired.

**Figure 2:**
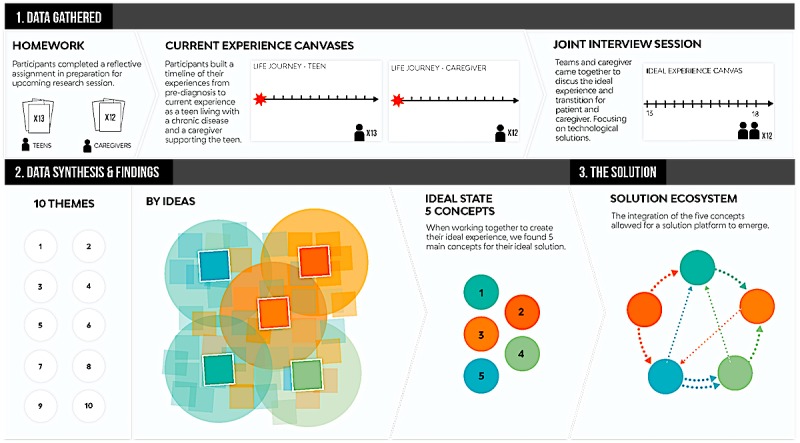
Outlining the process of data collection, analysis, and synthesis.

Figure 2 summarizes the data collection, analysis and creation of themes, and ecosystem process in this study.

### Analysis

#### Method of Analysis

As a part of the co-design process, the analysis was conducted manually (instead of using NVivo software). The data was captured during the interview on the canvases via visual tools, and Post-it notes, transcripts, and researcher notes were used as sources for the analysis process. To keep visual information in the analysis, we also followed design-based guidelines for on the wall analysis [9], a method that helps include visual materials from a highly qualitative interview into the analysis process.

Thematic analysis was employed to identify challenges, opportunities, and barriers that teens and caregivers faced at strategic times on their journey and the desired solutions [11]. Thematic analysis is a commonly used methodology to identify and analyze patterns within qualitative data and report the findings [12]. As defined by Braun and Clarke [12], thematic analysis includes the following progression: (1) familiarizing with the data, (2) generating initial codes, (3) searching for themes, (4) reviewing and refining themes, (5) defining and naming themes, and (6) reporting the findings.

#### Familiarizing With Data

To be familiar with the depth and breadth of the content, two researchers read the transcriptions multiple times while actively searching for meanings and patterns.

#### Generating Initial Code

Transcription texts were segmented and coded with notes using highlighters, colored pens, and Post-it notes. Then, they were grouped using similar terms and patterns. Codes were created by group decisions or consensus among the researchers.

#### Searching for Themes

Once all the data were coded, the codes were sorted and grouped into potential themes. To depict the patterns of codes and themes, a visual map was created. The description of themes and codes was used to clearly guide the grouping. The relationships between codes and themes were discussed among the researchers. Potential themes and subthemes were proposed at the end of this stage.

#### Reviewing and Refining Themes

All themes were reviewed to fit the code and content. If the theme did not fit, the researchers reworked the theme, created a new theme, or disregarded the theme. The transcriptions were reviewed again to be sure that the proposed themes fit and were not missing any codes or information within the transcripts.

#### Defining and Naming Themes

Themes were defined (what it is about and its content) and named with relevant terms in this stage. Similar themes were organized coherently and consistently according to their content.

#### Reporting the Findings

Themes were reported with sufficient content, supporting evidence, and examples.

## Results

### Themes: Challenges and Opportunities

The thematic analysis applied to the information collected from teens and caregivers resulted in 10 themes that identified challenges and opportunities during the transition to independent disease management. All patients and caregivers were frequent smartphone users, using at least 2 apps weekly. When talking about challenges, participants also spontaneously suggested some digital opportunities. Thus, we have also included self-reported technology solutions here.

#### Lack of Social Support and Communication

##### Key Challenges and Barriers

Teens and caregivers reported that in a crisis, they could not manage their condition without the understanding and help of others, (eg, teachers, school nurses, classmates, coaches, and extended family). However, others are frequently not informed on or misunderstand their condition or are ignorant about how to offer relevant assistance. Some parents realized the core issue.

Educating her [the teen’s] core group of friends was the best thing we could do for her because they know what she needs when mom isn’t around.

Epilepsy caregiver

Caregivers also reported that they often have contentious relationships with school nurses and administration. Some parents perceive that schools resist providing equal opportunities to children with chronic conditions.

When we got into the school district that we're in now, like, 3rd grade, they wanted to diagnose [my child] with ADHD...one of the teachers said, “You need to medicate your child.” And I said, “You need to learn how to teach my child.”

ADHD caregiver

In addition, tools that facilitate communication between HCPs, caregivers, and school nurses are needed to assist all health caregivers in managing updates in a teen’s care. Support in rural areas can be even more challenging. Families who live in rural areas have fewer support groups and resources available. Traveling nurses in rural schools are frequently not available. Thus, school nurses who may only be available one day a week are not a sufficient support system.

##### Digital Opportunities

Participants identified several technologies that could help fill in for their lack of support. This included (1) an audio, video, or text tutorial sent to the caregiver (eg, mom, school, nurse, or coach) on how to manage a health crisis; (2) a real-time instructional video to educate witnesses and friends about how to help in a crisis (when the crisis is happening); (3) prescription updates sent digitally to the school nurse; (4) tools that simplify coordination between the caregiver and school nurse; and (5) a tool that will give coaches or teachers real-time alerts or symptom-reading skills so that they can pull teenagers out of the game or class and ensure that they get help.

#### Managing Social Stigma

##### Key Challenges and Barriers

Teens did not want to be labeled as a sick person nor draw too much attention at school, which may lead to feeling isolated, ostracized, or depressed. Depression may also induce them to skip their therapy at school, no matter how urgently needed, to defend against being seen as different. Labels like “sick person” may cause peers to misconstrue a teen’s abilities and limitations and also lead to hurtful comments, judgments, and unwanted advice. These misconceptions disrupt developing trusting friendships that could provide needed support.

The first grand mal seizure happened in school in front a lot of her classmates, since then she feels awkward and ashamed of the condition because no one there gets what she has.

##### Digital Opportunities

Teens and caregivers suggested that education and communication technologies could develop or enhance peer awareness. To alleviate the social stigma that teens with chronic disease may face with peers, some teens suggested having a short video ready on a phone app to inform peers in simple language what the chronic diagnosis is and means, how it affects the diagnosed teen, and how peers can be respectful of the teens’ struggles with the disease. To encourage communications, a digital platform that facilitates sharing positive stories of older patients overcoming hurdles with younger teen patients to help overcome depression and anxiety was suggested.

#### Access to Education

##### Key Challenges and Barriers

Following diagnosis, caregivers are frequently frantic to understand the disease and its short- and long-term implications. The most common question asked is, “How will my child’s diagnosis and treatment impact their lifestyle and the family’s?” Caregivers are looking for the right information at the right time in the right dose. For instance, soon after their child’s T1D diagnosis, a caregiver joined a Facebook group hoping to find tips and support. They were devastated when other caregivers were discussing the potential long-term effects and worst-case scenarios. The group did not realize that someone new was present in the conversation. During the diagnostic and initial adjustment phases, caregivers are uncertain where to find trustworthy, authoritative sources of information and frequently get misguided or insufficient information.

You know, you turn to the Internet, parents of kids with diabetes, which is helpful in some ways with some practical questions but, in other ways, frightening because a lot of...I finally had to like exit because it was a lot of moms that were really just really scared.

T1D caregiver

No one was telling me anything...I mean I knew I had stuff wrong with me, but no one was telling me what it was or what it meant or what that means for the rest of my life.

ADHD Teen

##### Digital Opportunities

In their quest for knowledge, caregivers reported technological solutions that would help to facilitate access to the education they are looking for. This included precategorized discussion groups that allow the caregiver to match what they are looking for before they get caught up in topics that are not helpful and preapproved, credible information sources (eg, reputable clinics, local hospitals, their doctor) that provide access to reliable information when they need it the most (eg, when symptoms do not make sense and caregivers want access to tips). The caregivers did not want to browse through thousands of sites to find key information. Also, a tool that helps compare efficacy among competing manufacturers (eg, commercial options for medications or food brands in the case of diabetes) was proposed.

#### Symptom Monitoring and Support

##### Key Challenges and Barriers

The initial adjustment period is rife with triggers, which frequently catch caregivers and teens off guard and can lead to full-on symptoms. Without timely support, symptom onset causes significant distress of being overwhelmed or unprepared. Teens are looking for ways to get immediate help for their symptoms without having to call their caregiver or an HCP. Avoiding hospitalization is certainly another key goal for the family.

Caregivers and teens struggle with how to deal with sudden symptom onsets, especially at night, or when the teen is away from home or the caregiver. Both teens and caregivers are diligently seeking pre-emptive alerts of a health care crisis. For instance, one participant caregiver reported that she constantly wakes up in the middle of the night to double-check her teen’s blood sugar. A mother of another teen fears a sudden unexpected death in epilepsy crisis, known to occur overnight with people who live with epilepsy.

In addition to unmet needs regarding symptom monitoring, teens also desire actual symptom relief or advice when they are away from their caregiver or other support. Like this caregiver who had a son with ADHD and anxiety:

I [caregiver] said, “Are you really anxious about this?” And he [teen] said, ”Yeah. Why?” And I said, “Because you have scratches.” So, having an app or some sort of technology that could be like, ”Hey, you're kind of, digging your...a hole in your arm. Can't you stop doing that?” Like, it would be awesome. So, if he was able to be like, “Oh, I'm scratching. I should put that in. That's happening.” And so now he knows when it happens, and we can talk about what was going on when it happened.

###### Digital Opportunities

Participants outlined 4 technologies that would support them in symptom monitoring. First, a symptom trigger tracker that gives advance notice to the caregiver when symptoms may be developing. Second, voice-activated tools (like Alexa, Siri, or Google assistant) to orally report and record symptoms (eg, “begin timing of seizure”) or have a technology to automatically begin videorecording the episode for future reporting to HCP. Third, tools that capture potential triggers or patterns over time that are unique to each teen to prompt early responses or pre-emptive actions. Fourth, families dealing with diabetes, CF, and epilepsy particularly desire night monitoring in the form of a smartwatch, clothing, or wearable scanner. These devices could sound an alarm when vitals suggest a crisis is imminent or send automatic SMS text messages that warn and report to the teen, doctor, and caregiver, simultaneously.

#### Safety During Driving

##### Key Challenges and Barriers

Caregivers have a constant fear that their teen may have a dramatic medical event while driving. Caregivers mistrust their teen’s judgment if they are driving when an emergency occurs.

So, if something could alarm her to sit down and be safe, you know. And or appear where she's driving, and to stop. Stop the car, pull over. Don't go any further. And I wouldn't care what form it would be in, if it would be a piece of clothing, if it would be the watch, if it would be, you know, a kind of, like, an earpiece, like those things that people use, a…not a GPS, Bluetooth. That's it. Something to make her stop what she's doing.

Epilepsy caregiver

##### Digital Opportunities

Caregivers shared several technological solutions that may help to reduce risk when driving. First, technology to prevent teens from driving if they are at risk of a medical event because their symptoms are not under control (eg, similar to a breathalyzer that stops someone from driving under the influence). Second, tools that help avoid the event that causes the teen to lose control when driving, such as extremely low blood sugar, seizures, and difficulty staying attentive. Third, wearable technology, in jewelry, clothing, or a watch, similar to an emergency button in the car. When pressed, the device transfers key health information to first responders or other passengers. Last, technology that records the teens’ driving activity. Records can be used to differentiate if an accident was due to their condition or poor judgment.

#### Access to Health Care Providers

##### Key Challenges and Barriers

Issues accessing HCPs are numerous and varied. Key barriers include distance to hospital, scheduling problems (cancellation, wrong scheduling), responsiveness to requests and questions, lack of integration and consistency among multiple providers, and communication issues with or among HCPs.

[Doctors] don't listen to me. I called, I paged them that night because she still couldn't walk after two and a half hours. She had hit her head on the wall and then the floor, and I paged neurology after the first one and they were like, ”Oh, well just increase her meds.” They don't want to see her, they don't want to do anything else. They just want to increase her meds. I was livid.

Epilepsy caregiver

Patients and caregivers who need to travel long distances or frequent visitors with scheduling issues suffer the most, which leads to delayed health care services. Currently, communication with doctors is based on what the caregiver or teen can recall without written instructions. Further, no significant communication usually occurs between visits to HCP. However, teens are willing to text or communicate directly with their HCPs to bridge that gap.

##### Digital Opportunities

Five technological solutions were suggested. First, a 24-h SMS text message-based helpline to health care providers—not necessarily their own HCP—but an HCP they can trust and ask general questions. Second, a real-time decision-making tool to answer questions like “I am going to work-out for 2 hours. What snack may be good?” Third, a channel where the teen and caregiver can contact an HCP expert and get general advice for noncritical situations without waiting until the next HCP appointment. Fourth, caregivers need a preappointment tool so that teens can prepare questions before going to the appointment. Fifth, telemedicine to improve access to care.

#### Relationship Between Caregiver and Patient

##### Key Challenges and Barriers

The caregiver has a potential to be the “bad guy” since they take on the responsibility to remind the teen regarding proper management of their diagnosis. Because teens struggle with medication compliance, caregivers constantly remind teens to take medications. Conflicts over compliance may cause relationship problems. Some teens who feel overwhelmed with therapy may lie to their caregivers. Besides being detrimental to their therapy management, this situation may cause the caregiver to mistrust the teen.

Just when you think you got it...Everybody was really trying their hardest, and that's when I'm finding the meds being hid. And it's like, “I thought we had finally had a breakthrough and now defeated again,” back to square one.

ADHD Caregiver

##### Digital Opportunities

Communication technologies, which assume the reminder-police role, may help to increase the strength of the relationship between teen and caregiver. Caregivers wish that technology could deliver news, reminders, and directions, so they did not have to be the “nagger.” Teens want improved and remote communication between the caregiver and themselves when they first move out of the home (eg, college). A tool was suggested to allow the caregiver and teen to collaborate on a daily checklist that will help with reminders but also provide a way for caregivers to check in instead of verbally asking multiple times a day. Teens believe this tool will help improve their relationship with the caregiver. A tool that offers caregivers objective proof that the teen has done their therapy (eg, a vitals readout, video footage of the teen completing therapy-related activities) was also proposed. Lastly, a digital assistant tool was suggested that, when the patient desires, can give their caregiver a real-time readout of vitals or other statistics that will help to double-check decision making and make check-ins easy.

#### Long-Term Perspective

##### Key Challenges and Barriers

Caregivers perceived that their teen tends to think in the moment without considering how their actions and choices can have a negative impact on their therapy path. In addition to gaining life skills and understanding as any normal teen, teens with a chronic medical condition have the extra challenge of learning about the different circumstances caused by their condition.

I worry if my son understands that how the choices he makes now will affect his long-term health. He has his typical teen attitude of resisting our instructions, but it will have a big impact on his future

CF caregiver

##### Digital Opportunities

Technology to help develop a right mindset for better decision making was requested. This included decision assistance tools to help teens understand how choices today affect long-term health, technology to visually demonstrate how good choices today add up long-term, and scenario tools that help teens think through situations specifically related to their condition and prompt them to be proactive about proper management.

#### Supply Management

##### Key Challenges and Barriers

Teens do not want to be burdened with keeping track of their supplies (eg, ordering, and maintaining an emergency stash), especially when at college. Some teens did not have access to school nurses, as these nurses serve more than one school on any given day. Thus, when an emergency supply is needed, a backup is not available (eg, students with T1D at rural schools).

Parents expressed feeling overwhelmed managing the medication-supply aspect of care. In addition to managing their own busy schedules, they also carry the burden of keeping an inventory of supplies and medications. Families with several children or with a teen with multiple conditions face greater challenges in managing supplies.

I have a hard-enough time keeping up with all their school activities and appointments. It’s overwhelming to keep up with all the medications and making sure we have all the refills at the right time for each of my children.

Epilepsy caregiver

##### Digital Opportunities

Teens and caregivers shared their ideas about technology that could help such as inventory tools to assist in tracking and to alert caregivers when supplies are low. Another suggestion was smart ordering, delivery, and storage for a seamless ongoing supply, which could ease the transition to college or away from home.

#### Financial Struggles

##### Key Challenges and Barriers

Caregivers are commonly overwhelmed with complex insurance policies and health care coverages. While they suspect they may not have the optimal coverage, they lack time or expertise to compare or assess options. For example, despite long-term use, they may experience a sudden loss of access to a drug or an important medical supply. Solving this problem can be time-consuming and anxiety-provoking. Finances are not a teen stressor at this stage.

Insurance, we had to adapt to the Ohio systems. Well, it's the secondary insurance that was really the problem...I’ll just say, secondary state-assisted insurance, essentially, changed. This is very stressful.

CF caregiver

Furthermore, caregivers are aware that some medical technologies are limited by an insurance company’s willingness to pay and also because demand exceeds supply. They also wish to tap into the insurance navigation expertise they believe exists among the professionals at hospitals. In that regard, caregivers would like to rely on hospitals to give advice on getting the best out of their insurance or choosing the best insurance, considering the chronic conditions they have.

##### Digital Opportunities

Caregivers expressed their expectations on technology-based financial decision support. This included a digital concierge-type service to better compare options and maximize coverage and digital tools to improve the price transparency in care.

### An Ecosystem of Technology Solutions to Facilitate an Ideal Transition

In this study, once we identified the challenges in self-management throughout their life journey for both the teens and caregivers, we primarily used the participant’s inputs to develop recommendations for technological solutions and opportunities that could facilitate the transition to independence. Our patient-centered approach helped to identify some digital opportunities that could assist teens and caregivers in achieving an ideal transition. The following 5 solutions were synthesized from the ideal experience activity that the patient and caregiver collectively envisioned. To support each proposed solution, we also include examples from the current literature.

#### The Teen-Caregiver Communication

A new platform (eg, device, app, or software) that would provide support and cushioning in communication several times a day between caregiver and teen during early transition. This platform would act as a bridge by providing a collaborative, task-sharing platform with a built-in reward system. However, the platform should also evolve to help the teen expand their support network while keeping the caregivers in the loop through weekly or monthly reporting.

##### Current Implementations

Researchers from Michigan State University have proposed a mobile app, MyT1D Hero, to create a communication platform among teens with T1D and their parents to support self-management [7].

#### Education and Tracking

This integrated platform would provide a channel for a daily dose of age-appropriate education and information tracking for the teen and support caregivers in their daily decision making. The focus at the early stage is helping the teen understand their body, their disease, symptoms, and triggers. Later in the transition, when teens start making therapy decision on their own, the platform needs to evolve into becoming a coach.

##### Current Implementations

The gamification concept has been used to educate kids and parents about managing diabetes [13] and for tracking the teen’s condition [14,15].

#### Teen-Caregiver and Health Care Providers Communication Bridge

This platform needs to provide seamless communication between HCP, caregivers, and school to lessen the additional burden on caregivers and keep everyone on the same page. The teen begins to communicate more with their HCP early on to help build trust, while the caregiver continues to be the main point-of-contact and influence in the early stage.

##### Current Implementations

A communication platform among caregiver, HCP, school administration, and the teen has not been observed. However, the use of social media was found to be effective in creating a communication network, but not without several limitations and privacy concerns [16].

#### Emergency Support System

This platform would provide emergency support and cautionary alerts for caregiver and teen and external networks (eg, HCP, school nurses, and first responders). The system needs to be designed to prevent serious consequences from the sudden onset of medical events from occurring, while also training the teen and peers to know how to act and respond during an emergency. This system would be connected to a mobile device (phone or wearable device) for the teen that is similar to a medical alert button.

##### Current Implementations

Emergency support systems at the individual level for teens have not been observed in CDM literature. However, emergency apps available in the market can be leveraged in chronic disease-related emergencies, such as Medical ID app [17].

#### Supply Management System

This platform aims to reduce the burden of supply management and organization for the caregiver and teen by providing a fully integrated system of alerts, reminders, and automated supply replenishment and management. Additionally, the platform could provide assistance with and education regarding insurance and financial and legal support.

##### Current Implementations

Although integrated supply management has not been observed in the literature, Mango Health app [18] enables caregivers and teens to track their medications. Retail pharmacies like CVS and Walgreens are also starting to have integrated systems for patient’s medical supply management.

**Figure 3:**
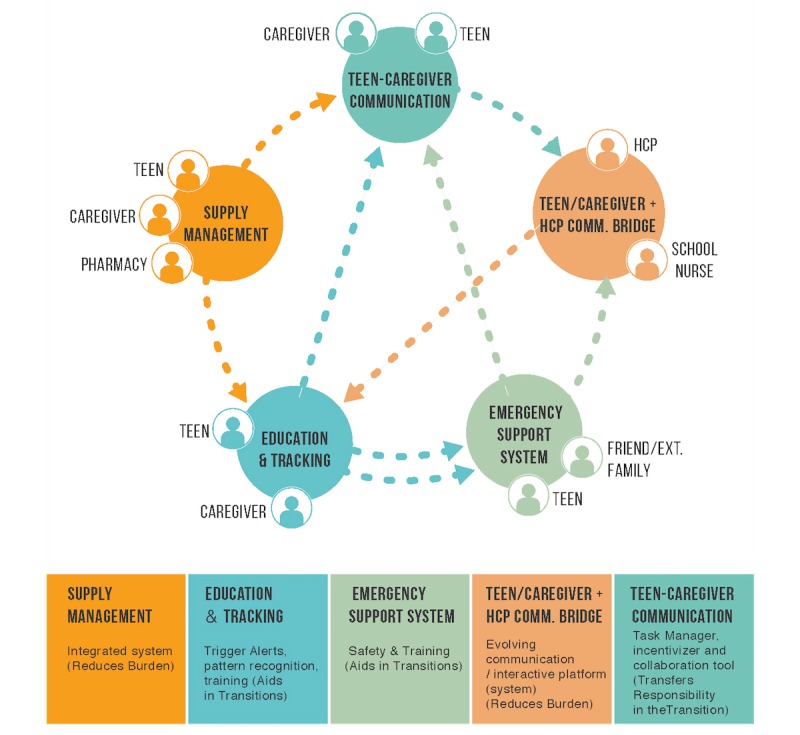
Outlining the proposed ecosystem of digital health solutions. HCP: health care providers; comm: communication; ext: extended.

#### Proposed Integrated Ecosystem of Digital Health Solutions

The five technology opportunities above are interrelated and form a technological ecosystem, enabling information flow and communication between the solution systems to create an integrated approach to CDM. The proposed solution ecosystem (Figure 3) is designed for seamless communication and information flow among parties and technology solutions to assist a teen in managing a chronic disease during transition to independence. In the figure, the circles represent the 5 digital opportunities identified to enhance the teen-to-independence transition while managing a chronic diagnosis. Lines in between the concepts indicate integrated lines of communications between other digital solutions. These connections are hypothesized based on study observations. The boxes present examples for each solution. Each of these solutions can evolve with the changing needs of the teen throughout their transition. For instance, the teen and caregiver bridge may promote medication compliance in early transition, logging therapy tasks and sending reports to HCPs in midtransition, and using time management tools for managing therapy in late transition. Further interview notes on technology opportunities in transition to independence are provided in Multimedia Appendix 2.

## Discussion

### Transition Challenges Require a Multifaceted Solution Approach

The ten themes identified in our study are well supported by the literature. The first two themes “*Lack of support and communication”* and “*Managing social stigma”* revealed that support from the social environment is a fundamental need, necessitating a deeper understanding of teens’ social state [19]. Koetsenruijter et al [20] also argued that individuals with chronic conditions need social support for health management.

In the third theme “*Access to education,* ” to support their teens, caregivers desire *access to educational material* to learn as much as possible regarding the disease’s symptoms, management, and treatment options. Despite the importance of education to support self-management in CDM [5,19], the literature suggested that barriers to having quality education and information included unreliable Web resources and limited health and computer literacy of users [21].

In the fourth theme “*Symptom monitoring and support*,” in addition to managing symptoms, families want a pre-emptive advantage through continuous *symptom monitoring*. If an impending medical crisis could be identified through early alerts, their teens could receive aid quickly, possibly mitigating more severe consequences. Several digital solutions have been offered for symptom monitoring and support [22], yet adoption is not widespread.

The fifth theme “*Safety during driving,* ” that is, *safety of teens while driving* is another major concern. Since commuting is a significant part of the daily routine, caregivers are rightfully concerned about medical emergencies that may occur while teens are driving. The severity of driving accidents among young drivers with a chronic condition has been argued in the literature. Comparing young drivers with and without ADHD, a study demonstrated that teens with a chronic condition had higher driving risks [23]. Thus, CDM initiatives should incorporate safe commuting.

The sixth theme “*Access to health care providers”* is problematic regarding open communication and information sharing. CDM technologies, which are available to access health care, are highly efficient and help reduce clinic visits [5], but our findings suggest that the practical use of these technologies has not yet reached maturity.

Likewise, technology use in families revealed that communication technologies would enhance a reciprocal *relationship between the caregiver and teen*, which was the seventh theme. This would be a promising aspect of technology use, to further extend the benefits of technology (eg, mHealth interventions), in health care management and communication for teens among caregiver and HCP [4].

Communication technologies can also be used to support teens making healthy decisions from a *long-term perspective,* the 8th theme. Clinical decision-making systems have been proven successful in CDM in a clinical environment [24]. Yet, our findings suggest that the focus for decision support needs to be individualized for teens to assist them in transitioning.

Similarly, individualized technologies for controlling medicine inventories and enhancing personal *supply management,* the ninth theme, would assist teens in transitioning to independence.

Above all, caregivers report *financial struggles,* the tenth theme, as a major barrier to accessing current assistive technologies in CDM. Thus, financial issues may have a mediating effect on other challenges. Likewise, the families interviewed wished to receive medical support using low-cost communication and information technologies.

### Guidance and Empowerment Through an Ecosystem of Digital Solutions

A single technology solution was insufficient to meet the many challenges patients and caregivers face in launching to independence a teen with a chronic condition. Rather, our study derived an ecosystem of digital health solutions. The 5 proposed technology opportunities for ideal transitioning were derived from self-reported technological expectations of teens and caregivers. Fundamentally, these opportunities reflect their expectations and need for a communication system that links the core stakeholders (patient, caregiver, and HCP).

Measuring the patient’s quality of life and quality of communication among caregivers, teens, and HCPs is problematic [5,19]. Therefore, to overcome the major communication issues, we propose developing a communication platform. Similarly, enhancing medical education and health literacy would also be beneficial during transition to independence and for the long-term CDM of teens [5]. The identified opportunities align well with Miller et al’s [25] findings regarding the technology preferences of young people in transition for access to health care and communication needs, and Ranade-Kharkar et al’s [26] information goal types among HCPs and caregivers for kids with special health needs.

From a broader perspective, teens may benefit from using technology in the long term, starting with the early introduction of technology tools and successfully engaging with technologies through adulthood for CDM [27,28]. The technology used for communication and self-management would facilitate the treatment and consulting process, assist teens with condition-specific needs, and make digital CDM more sustainable [5,19].

Still, as per the suggestions in this study, sustainability and long-term engagement need focus and familiarization to reduce teen frustration and reluctance with technology [29,30]. In that regard, Griffiths et al [6] suggested using technology-based health care services with an existing, trusted HCP team for conveying services to identified needs. The HCP team also needs to work on effective information resource use for timely access. To maximize efficacy, collaborative co-design with patients and continuous improvement of solutions should be considered [2]. Within this context, gamifying the CDM concept to promote engagement, sustainable self-management, and communication is another possible approach for digital health development.

### Limitations

As a quality improvement study to improve digital health delivery at NCH, we recognize important limitations on generalizability. Our study covered only 3 of the 9 top chronic conditions among children in the United States [31]. Also, the sample size may be insufficient to derive generalizable results. Since the study lacks quantifiable input, power and other statistical analyses are precluded from testing our findings. As is frequently the nature of qualitative methods, analyst bias could have affected this study to some extent in both data collection and interpretation. These limitations can be addressed by future implementations to validate the findings from this study.

### Conclusions

In this study, challenges and barriers for teens with chronic diseases and their caregivers were identified, discussed, and matched with technological opportunities and solutions. Technological solutions and digital health mechanisms were suggested as mediating tools for better communication among patient, caregiver, HCP, and authorities. These findings would help to extend current efforts using mHealth management and intervention methods [32]. We suggest future studies to create a virtual bridge between individuals and institutions and to disseminate the technology and its use.

## Appendix

Multimedia Appendix 1 Tools and materials for the data collection activities.

Multimedia Appendix 2 Interview notes on technology opportunities in transition to independence.

## Abbreviations

ADHD: attention deficit hyperactivity disorder
CDM: chronic disease management
CF: cystic fibrosis
HCP: health care provider
mHealth: mobile health
NCH: Nationwide Children’s Hospital
T1D: type 1 diabetes
