# Supplementary material for: Proposing an Ecosystem of Digital Health Solutions for Teens With Chronic Conditions Transitioning to Self-Management and Independence: Exploratory Qualitative Study
Source: J Med Internet Res. 2018 Sep 6;20(9):e10285. doi: 10.2196/10285 (PMC6231785; doi:10.2196/10285)

Multimedia Appendix 1. Tools and materials for the activities

Assignment

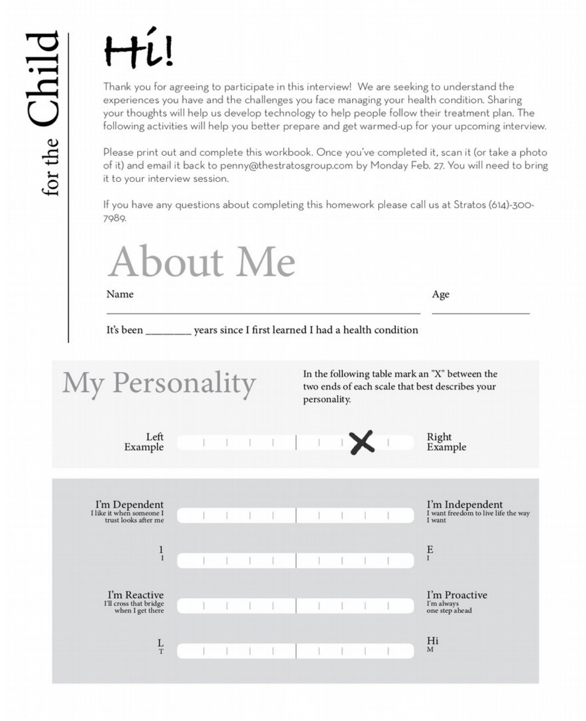

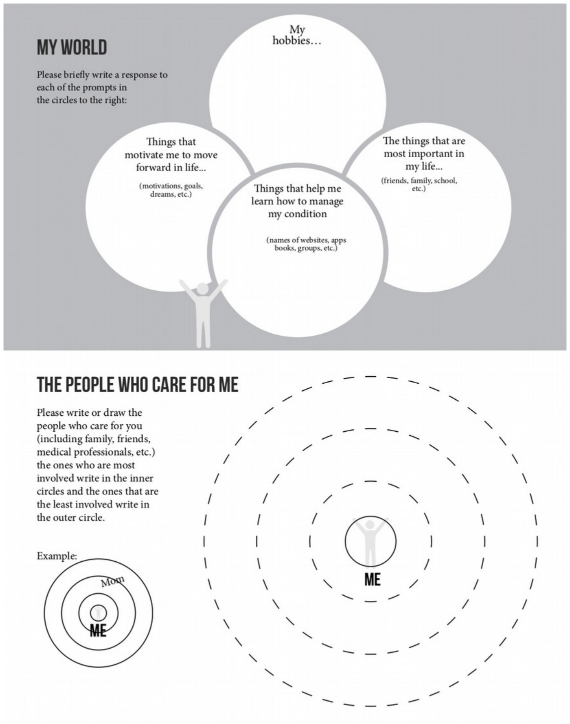

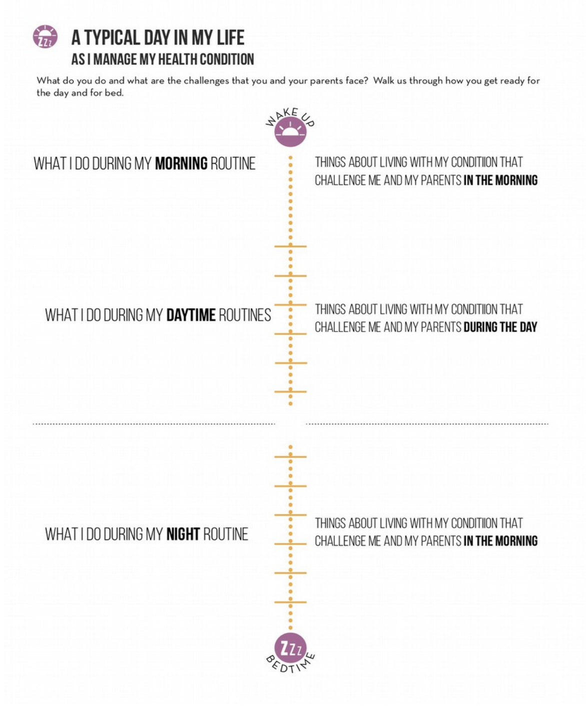

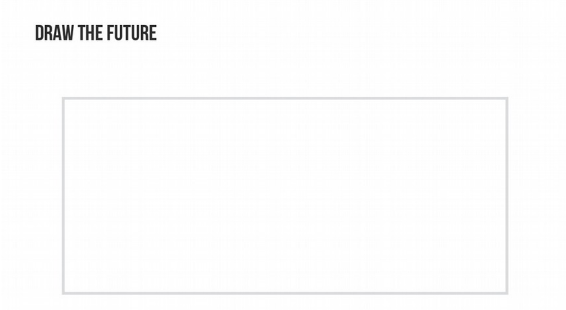

Activity 1

Canvas, materials and a sample of finalized activity

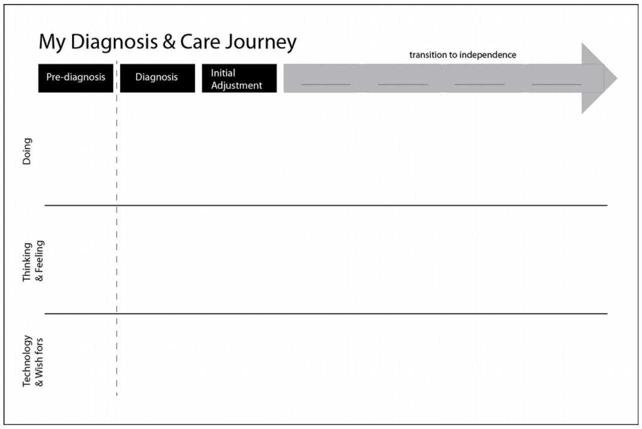

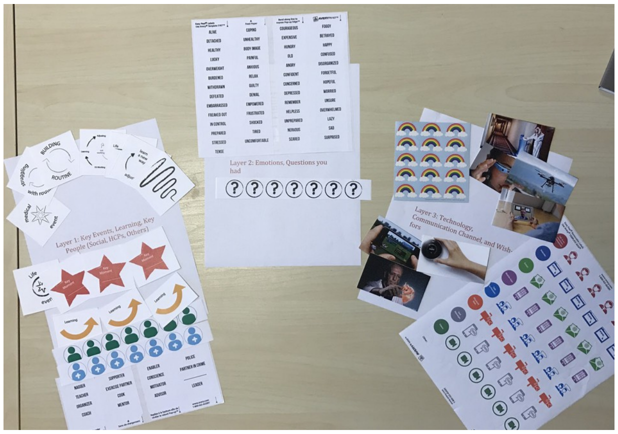

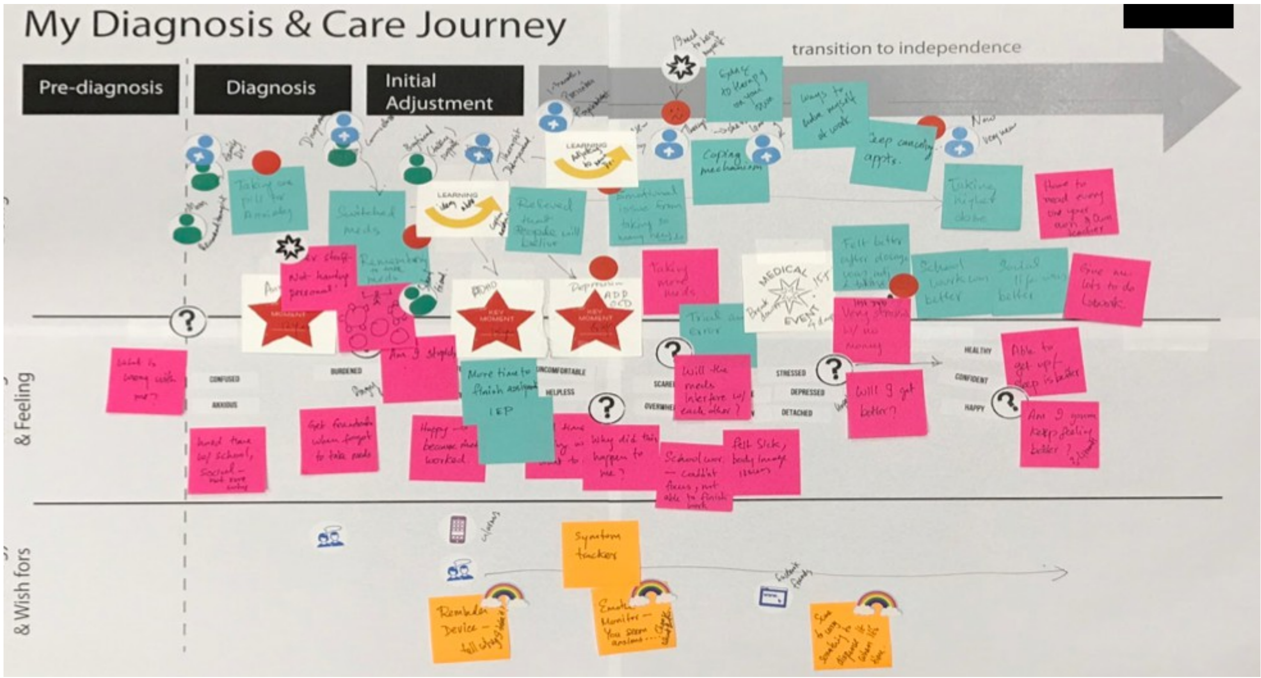

Activity 2

<u>Canvas, materials and a sample of finalized activity</u>

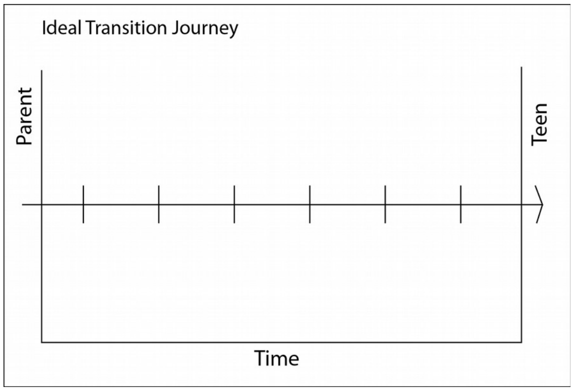

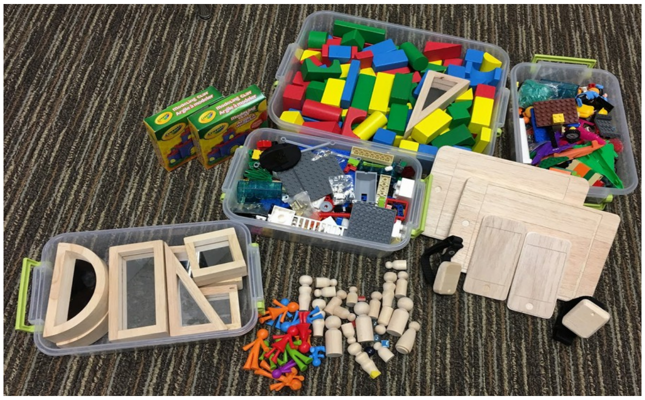

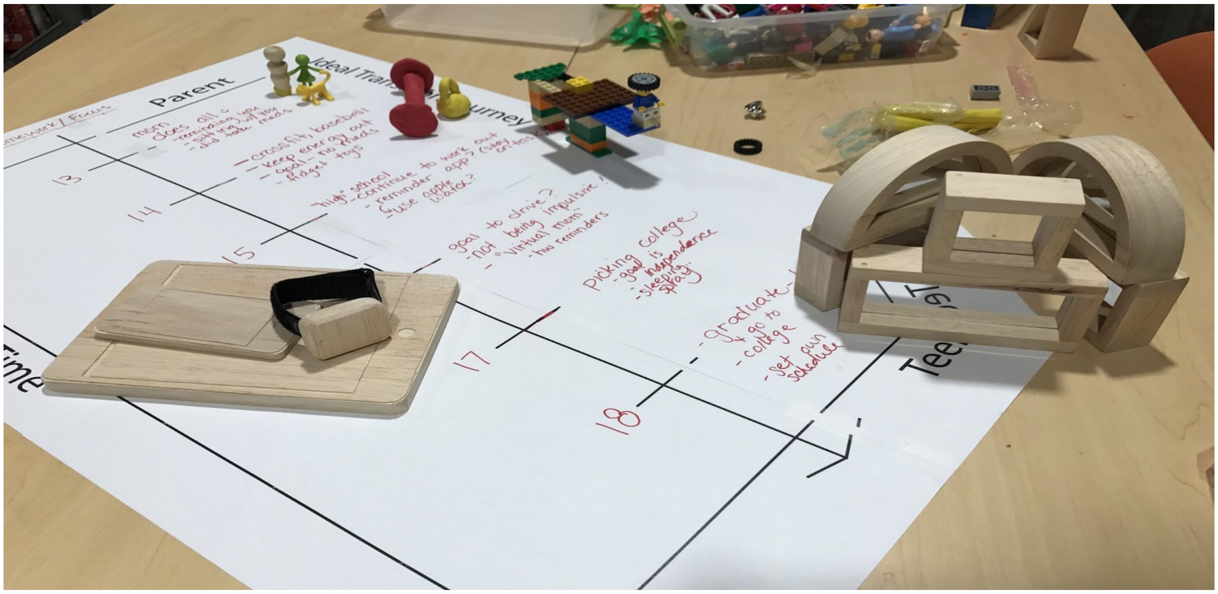

Supplement: Multimedia Appendix 1 [file jmir_v20i9e10285_app1.pdf]
