# Supplementary material for: Proposing an Ecosystem of Digital Health Solutions for Teens With Chronic Conditions Transitioning to Self-Management and Independence: Exploratory Qualitative Study
Source: J Med Internet Res. 2018 Sep 6;20(9):e10285. doi: 10.2196/10285 (PMC6231785; doi:10.2196/10285)
Supplement: Multimedia Appendix 2 [file jmir_v20i9e10285_app2.pdf]

Multimedia Appendix 2. Technology opportunities for teens in their transition to independence

**Transition stage/ Ecosystem**	**Early**	**Mid**	**Late**
**Teen- caregiver communication**	Has a collaborative checklist that caregiver and teen can adjust depending on what transition phase they are in.<br><br>Shows proof of medication compliance, therapy and school tasks to caregiver.<br><br>Enforces grounds rules (e.g. for ADHD, turn off tech at bedtime).<br><br>Connects and gives reminders through a smartwatch and/or something like voice activated device.<br><br>Has a positive reinforcement reward system customized to child's motivation style.	Create goals toward independence and milestones to achieve.<br><br>Logs therapy tasks and sends report to HCP.<br><br>Shows milestones for stages of transition to independence.<br><br>Teen users can expand their care network and find other teens with similar or same condition.<br><br>Shows task completion, including countdown.<br><br>User can log in goals they've achieved.<br><br>Users can track the building of routines.	Teen can add friends or other family members to the care network.<br><br>Monthly report on mood, school and friends.<br><br>Time management tool for teen to manage therapy and other responsibilities.
**Education and tracking**	Learn about body, the disease and how the condition is affecting them.<br><br>Have short videos teen can share with peers to minimize myths and bullying.<br><br>Tools to identify triggers and how to avoid complications.<br><br>Monitor symptoms and triggers at night time which can alert caregiver if something goes wrong.	Learn about medications and side effects.<br><br>Log and send symptom tracker info to HCP or caregiver.<br><br>Scenario building feature to help teens think ahead for new situations.<br><br>Provides coping skills and tips.<br><br>Provides job management and career	Algorithm sees trends in tracking data over time and can give advice on triggers and symptoms.<br><br>Shows spikes and patterns in symptoms for the HCP and caregiver to create new strategies.<br><br>The technology becomes a coach for the teen.

	Gives caregiver support when to set up appointment with HCP based on severity or new symptoms.<br><br>Review testimonies or stories of successful management of other people.	advice from someone with the same condition.	
**Teen/Caregiver + HCP bridge**	Has reminders for doctor’s appointments.<br><br>Caregivers asks relevant questions to HCPs wherein they can tag the urgency level.<br><br>Gets auto authorization for medicine changes from HCP.<br><br>Makes it easy to transfer info to a school nurse (e.g. changes in treatment plan, medication, mood changes).<br><br>Caregivers can send notes about the child’s condition to a teacher, substitute teacher, school nurse, or coach. Have ability add or remove care team members.<br><br>Sends medication interaction alerts between HCPs.	Allows teens to login at 16 or 17 yrs old.<br><br>Has Facetime/text for quick assistance with HCP so student doesn’t miss school.<br><br>Prepares teen/caregiver for appointments. Tool allows teen to input questions and update before the appointment and start communications directly with HCPs.<br><br>Reminder to make appointments via integration with voice activated devices and hospital apps.<br><br>Shows synced calendar views and time options for caregivers and doctor to select appointment time.	Teen sets up appointments and can do independent check-in with HCPs and get medication refills. Sends summary reports to caregiver if teen does independent HCP appointments.
**Emergency support system**	In emergency situations, displays contact information for caregiver.<br><br>Prompts questions to hinder impulsive decision making by teen.<br><br>Connects person(s) in care network automatically during a crisis.	Provides early alerts on symptoms so person has time to stop what they are doing and get to a safe place.<br><br>Transfers all medical information to emergency responder system.	

	When teen is having a sudden medical event (e.g. seizure/hypoglycemic incident) it plays a video (on watch or phone) so bystanders know what to do		
**Supply management system**	Has a medication countdown system.<br><br>Is organized by each child in the family.<br><br>Sends auto refill notifications to the pharmacy.<br><br>Includes delivery service for medication, including the specialized medicines from NCH.<br><br>Connected to school supplies system to track the medication supplies they may need.<br><br>Connected to personalized concierge service to help with the insurance, financial and legal support.	Teaches the teen about medications names, uses and interactions.<br><br>Notifies teen when medicine is running low, which increases awareness of the importance of supply management.	Can transfer administrative access to teen at age 18 to manage the system and take over when they leave home for college.
